# Type 2 Diabetes Patients’ Views of Local Pharmacists and Fulfilment with Pharmaceutical Diabetes Care in Syria’s Latakia Governorate: An Online Survey Research

**DOI:** 10.3390/healthcare11121720

**Published:** 2023-06-12

**Authors:** Sarah Al Assaf, Dénes Kleiner, Romána Zelkó, Balázs Hankó

**Affiliations:** University Pharmacy Department of Pharmacy Administration, Semmelweis University, 1092 Budapest, Hungary; sarah.assaf@phd.semmelweis.hu (S.A.A.); kleiner.denes@semmelweis.hu (D.K.);

**Keywords:** type 2 diabetes, community pharmacists, diabetes pharmacy services, Latakia, Syria

## Abstract

Assessing the attitudes of diabetic patients towards community-pharmacy services and determining the demand for new services could help monitor and evaluate the therapeutic response. This study aimed to evaluate type 2 diabetes patients’ satisfaction regarding pharmacy care in community pharmacies and shed a light on the reasons for diabetic patients’ non-adherence to treatments. An online survey was conducted on a random sample of patients (*n* = 196) at the national Diabetes Centre in Latakia, Syria, from April to November 2022. The questionnaire consisted of four primary parts: (1) demographic characteristics of responders, (2) patients’ therapeutic behaviors, (3) diabetes knowledge, and (4) the general level of satisfaction with pharmacy diabetes services. The data were analyzed using descriptive analysis. Around 89% of respondents were satisfied with the information provided by community pharmacists. The patients’ non-adherence showed a maximum as a function of the number of concomitantly taken medicines, which indicated that in most serious cases patients’ adherence was increasing. Overall, most patients were delighted with community pharmacists’ expertise and pharmacy services. This positive image allows pharmacists to expand their duties as healthcare providers in diabetes care, and increase the patient therapeutic adherence by performing a reconciliation of the patient’s medicines, which involves reviewing all patients’ drugs and identifying realistic solutions to their adherence issues.

## 1. Introduction

Diabetes mellitus is the most common chronic and non-communicable disease afflicting people living in developed and developing nations [1]. A precise individual self-care performance is necessary for the effective management of this illness, including adherence to prescribed medicines, control of blood-glucose levels, a healthy lifestyle, and an improvement in health literacy regarding the many forms of this chronic illness [2]. Approximately 95% of people with diabetes have type 2 (T2DM). It frequently occurs in middle-aged and older people [3]. By the year 2045, 783 million individuals worldwide are expected to be affected by this illness [4]. Unfortunately, about half of these individuals will be unaware of their health condition. Effective solutions to this issue are required as it would place a major financial load on healthcare systems worldwide [5]. As reported by the International Diabetes Federation (IDF), the number of people with diabetes is predictable to increase by almost 39% by 2030 and by 96% by 2045 in the Middle East and North Africa. In Syria, in particular, the prevalence of diabetes is expected to reach 16.4% by 2045 [6]. Nevertheless, little progress has been made in addressing the problem [5,7]. Due to the ongoing conflict, Syria’s healthcare system has been destroyed over the last ten years, which has also had an impact on drug manufacturing. The lack of oral anti-diabetic drugs has a substantial impact on patients suffering from long-term conditions notably those from lower socioeconomic backgrounds [8]. Additionally, the inability of diabetic patients to follow recommended diets in conflict-affected nations is a barrier to treatment sustenance because diabetes medications must be taken with meals [9]. Healthcare in Syria is primarily funded by the government, while outpatient consultations and medications are frequently paid for out of pocket [10]. There is no universal health insurance program in place to provide care for all citizens. However, health insurance is offered to employees of public entities, some ministries, and professional groups [11]. Government health insurance covers the diagnosis and treatment of diabetes and cardiovascular disease, but not the cost of some chronic disorders such as Alzheimer’s or Parkinson’s [12]. Healthcare systems continue to face difficulties with pharmaceutical accessibility and availability. Major obstacles to the use and compliance with necessary chronic diseases exist worldwide but are particularly severe in low- and lower-middle-income nations. Those with chronic conditions such as diabetes have confronted multiple obstacles during the conflict, including insufficient access to medicines and testing supplies, as well as food shortages [13]. An expanded role for pharmacists in the healthcare system has been recognized by international standards for diabetes and is well-suited to this situation. In addition to dispensing medications, pharmacists offer diabetes patients assessment, education, monitoring, and behavioral counseling [14,15,16]. Aside from blood-sugar control, the primary goal of diabetes treatment is to prevent numerous complications that are the reason for increased mortality or substantial reductions in the quality of life for these patients. Although the use of medications helps to manage the HbA1c goal, a unique multidisciplinary team approach is also needed. Enhancing the effectiveness and efficiency of diabetic care requires the support of doctors, pharmacists, and nurses [17,18]. On average, a person with diabetes is estimated to visit the pharmacy three to eight times more frequently than other patients [19]. Therefore, community pharmacists have a chance to play a crucial part in controlling diabetes and its consequences by offering people strategies for tracking therapeutic interventions, promoting medication compliance, and counseling on lifestyle factors to enhance their quality of life [20]. Earlier studies [14,15,21,22,23,24] have demonstrated the pharmacist’s expanded role in the treatment of diabetes. In addition to this expanded role, pharmacists can also educate patients, refer them to a doctor when they have a disease or a drug issue, and check their medication consumption [14,24,25,26,27,28,29,30]. To fulfill these functions, four basic approaches can be distinguished: counseling, education, behavioral support, and a combination of the three. These techniques aim to influence patient treatment behavior, notably therapeutic adherence. With the provision and administration of medication, counseling services encourage both patients and pharmacists to have perspectives, viewpoints, and attitudes in a variety of scenarios [31].

Taking medications as prescribed is critical for reducing the unfavorable long-term complications of chronic diseases. A comprehensive study found that community pharmacists could play a key role in the management of T2DM by helping patients meet their therapeutic and lifestyle goals [21]. According to a Japanese study, individuals with T2DM who received lifestyle-change interventions from community pharmacists were able to maintain improved glycemic control [32]. With the growing number of T2DM patients in Syria, it is critical to explore the perspectives of these people toward community pharmacists, and to provide information about preferred pharmacy services and how such services can be enhanced to reduce the burden of diabetes. Therefore, the current study’s goal was to analyze T2DM patients’ perceptions of community-pharmacy services, with an emphasis on diabetic therapeutic adherence to help patients stay committed to their medications. Additionally, this study might shed light on the varieties of pharmacy services that Syrian patients should be able to access to enhance their quality of life.

## 2. Materials and Methods

### 2.1. Study Design

The current study was part of a Ph.D. dissertation inspecting the perceptions of Syrian T2DM patients toward community-pharmacy services. An online survey questionnaire was conducted over 8 months from April to November 2022 in the Diabetes Center in Latakia Governorate, Syria. The ethical committee of Al Manara University approved the study (approval protocol code 5245-2022).

### 2.2. Survey

A comprehensive literature search was conducted before creating a self-administered questionnaire [21,23,33,34,35,36,37,38,39,40,41].

The survey consisted of four components and is presented in the Supplementary Materials. Part 1 of the survey contained questions about demographic information (age, gender, place of residence, and education). Part 2 was composed of questions regarding patients therapeutic behavior (problems with daily medications, forgetting to take medicines, and number of drugs prescribed). In addition, for assessing therapeutic adherence, questions were adapted and modified from established scales such as the Morisky Medication Scale and the Medication Adherence Rating Scale (MARS), and then translated into Arabic [42]. Part 3 of the questionnaire addressed diabetes health status, covering the duration of diabetes, family history, and the possibility of having diabetes complications. Part 4 explored T2DM patients’ perceptions of community pharmacists’ capabilities.

Participants were informed about the voluntary nature of their participation in the survey, and they were assured that their responses would be used solely for scientific purposes. The questionnaire explicitly stated that only patients taking oral anti-diabetic drugs were eligible to complete the survey. Furthermore, it was emphasized that all responses would be recorded anonymously. Participants were also notified that answering every question in the questionnaire was mandatory.

### 2.3. Development of the Questionnaire

The survey was created in English by Sarah Al Assaf (S.A.A.), and the head of pharmacy administration Romana Zelko (R.Z.) reviewed it to ensure that the information was legitimate and appropriate. After that, Kind Darwish (K.D.) a second academic in the clinical pharmacy department who is a subject-matter specialist in the area of the present study examined it after being translated into Arabic. A pilot study was conducted before the actual survey of 13 patients to verify that the forms were comprehensible [43]. The survey took an average of 10 min to complete and was intended to be distributed online via Google Form (Google LLC, Mountain View, CA, USA).

### 2.4. Study Participants

We included only T2DM patients who took oral anti-diabetic drugs. No racial or gender restrictions were made. In terms of age, we only included adults between 18 and 70 years of age. The study’s sample size was estimated by referencing similar research. [23,24,32,33,34,35,36,37,40,44,45,46,47]. We excluded patients who took insulin and pregnant women.

### 2.5. Ethical Approval

The study was conducted following the Declaration of Helsinki and approved by the Ethics Committee of Al Manara University (protocol code 5245-2022). All participants included in the study gave informed consent.

### 2.6. Data Collection and Analysis

Excel software was used to extract survey data and convert it to a table (Microsoft Excel 2010, Microsoft Corporation, Washington, DC, USA). Statistical calculations were performed using SPSS 27 (IBM, Armonk, NY, USA) and Microsoft Office Excel 2016 (Microsoft Corp, Redmond, WA, USA). For statistical analysis, descriptive statistics such as mean and percent were used. For categorical variables, the results were shown as percentage-based numbers or as graphs.

The response rate was calculated from the newly registered patients and those who took part in our survey. The data in this survey are reported as percentages (%).

As there was no probability sampling, we only performed basic statistics and calculated raw numbers and percentages. Intended non-adherence was calculated from four questions (“Have you stopped taking your diabetes medicine(s) because you are suspected of feeling ill from taking this medicine(s)?”; “Have you stopped or discontinued your diabetes medication(s) because your condition has not improved and you are discouraged from taking the medication(s)?”; “When you feel that your diabetes is in balance, do you stop taking the medicine(s) for your diabetes?”; “Do you stop taking your diabetes medicine(s) if your friend/relative/neighbor is taking the same medicines as you and has experienced any side effects?”). If a patient answered yes to any of the questions, this person is defined as non-adherent. Overall adherence was calculated in the same way, but the answer to “Do you often forget to take your medication?” was also assessed.

## 3. Results

### 3.1. Characteristics of the Respondents

A total of 196 patients agreed to participate in the study. The response rate was 24.4% as, in the questionnaire timeframe, there were 803 newly registered patients taking only oral anti-diabetic drugs (as informed by the Diabetic Center). Most participants were male (55.6%).

Around 44.4% of respondents fell in the 46–60 age group. The participants had different education levels and half of the respondents (50%) were intermediate education holders. Regarding the diabetes health status of the respondents, nearly all of participating patients (89%) had had diabetes for more than 6 months and had a diabetic family member (73.5%). More than half of the respondents (62.2%) had a mix of comorbid conditions, which are risk factors for diabetes. Hypertension and high cholesterol levels were the most prevalent among comorbidities with (52.6%) and (36.7%), respectively.

Details of the demographic and patients’ characteristics are shown in Table 1.

### 3.2. Evaluation of Patients’ Adherence to Prescribed Medications

When assessing the daily medication routine, the majority of respondents (63.8%) indicated that it was not a problem for them. However, a significant portion (31.9%) expressed that they do encounter difficulties with their daily medication routine.

In terms of receiving recommendations from their general practitioner (GP) regarding diabetes treatment, the vast majority of participants (99.5%, *n* = 195) agreed that they received adequate guidance from their GP.

The relationship between patients and healthcare providers, including GPs and pharmacists, was reported to be positive. More than 90% of participants stated that they did not face any issues when asking their healthcare providers about their diabetes treatment, as shown in Table 2.

When asked about reasons for non-adherence, (25.0%) of participants admitted that they sometimes forget to take their medication. Additionally, (21.9%) reported that they would stop or discontinue their treatments if they felt their diabetes was under control, while (18.9%) would discontinue treatment if their condition did not improve. Furthermore, (17.9%) of participants mentioned that they would stop their treatments if someone around them suspected toxicity from the same medication, and 12.8% would refrain from taking their medication if they believed they were experiencing side effects.

The data provided in Table 2 shed light on the participants’ perspectives and attitudes toward medication adherence and the factors that influence their treatment decisions.

### 3.3. Percentage of Non-Adherent Patients Depends on Educational Level

**Figure 1:**
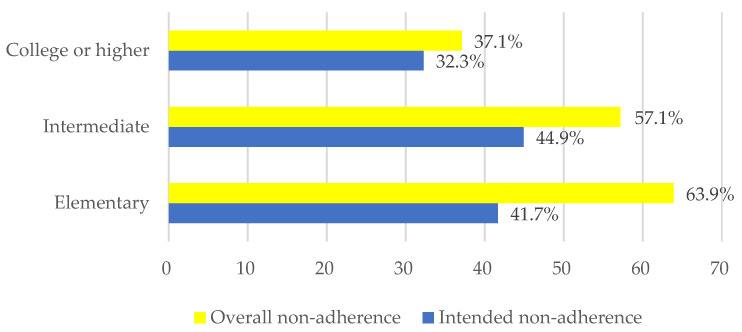
Percentage of non-adherent patients depends on educational level.

According to the data presented in Figure 1, the participants’ educational level appeared to be associated with their rates of overall non-adherence and intended non-adherence.

The elementary educational group had the highest overall rate of non-adherence, 23 from 36 (63.9%); while the college or higher group had the lowest, 23 from 62 (37.1%). In the case of intermediate education, 56 of 98 patients were overall non-adherent (57.1%), however, the highest level of intended non-adherence was seen in this educational level, (44 from 98 (44.9%)). On the other hand, only 15 and 20 patients showed features of intended non-adherence in the elementary and the higher educational level, respectively (Figure 1). All in all, the overall non-adherence was 52% and the intended non-adherence was 40.3% in the whole answering group.

These findings suggest that there may be a relationship between educational level and non-adherence behavior. It is important to consider educational background when developing interventions and educational programs aimed at improving treatment adherence among patients with diabetes. Tailoring interventions to address the specific needs and challenges faced by different educational groups can help promote better adherence and ultimately improve patient outcomes. Details are shown in Figure 1.

### 3.4. Reasons of Not Taking the Medications as Recommended from Health Provider

Based on our literature review and the recommendations for further research on the causes of non-adherence, we included questions in our survey to explore the main justifications reported by participants for not following their healthcare-provider’s recommended treatment.

**Figure 2:**
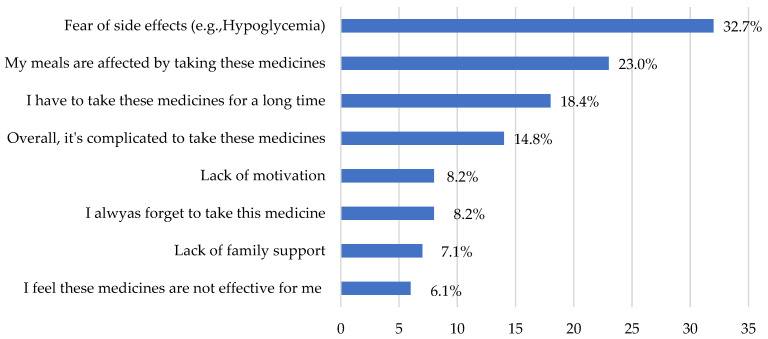
Common reasons that make patients stop their treatment.

Figure 2 presents the responses regarding the participants’ main reasons for non-adherence. The data clearly indicate that the fear of side effects was the most common reason reported, with (32.7%) of the respondents indicating this as a significant factor that could lead them to discontinue their treatment. This finding highlights the importance of addressing patient concerns and providing adequate information about potential side effects to alleviate fears and promote treatment adherence.

The second most common reason reported was the impact of the medication on meals, with 23.0% of the participants indicating that it affected their meals. This suggests that the practical aspects of medication administration, such as timing or dietary restrictions, can influence adherence behaviors. Understanding these challenges can help healthcare providers develop strategies to minimize the impact on patients’ daily routines and improve treatment adherence.

### 3.5. Concern

To assess the patients’ knowledge about diabetes and their understanding of appropriate actions to take when their health situation worsens, we included a question in our survey. The participants were asked to identify the solutions they would consider in such a scenario.

According to the survey results, more than half of the participating patients (52.6%) indicated a preference for visiting a general practitioner (GP) if they felt their health situation was deteriorating. This suggests that seeking medical advice and consultation from a healthcare professional is the most common choice among the respondents.

**Figure 3:**
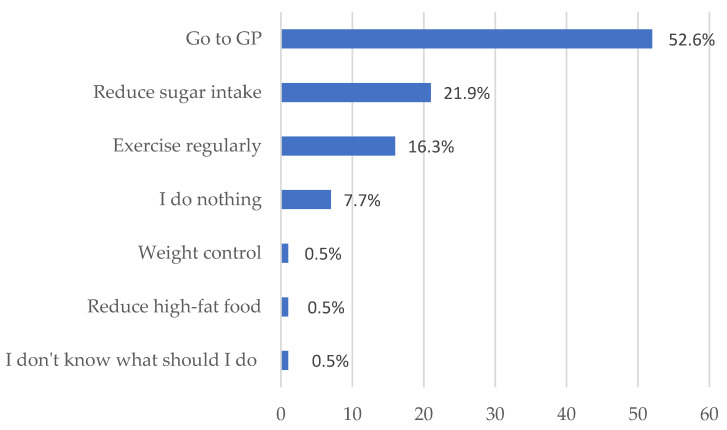
Patients’ behaviour towards health conditions.

However, it is important to note that a very small percentage of participants, less than 1%, mentioned strategies related to self-management and lifestyle modifications. Specifically, only 0.5% of the respondents reported that they would focus on controlling their weight and reducing their intake of high-fat foods. (Figure 3).

### 3.6. Percentage of Non-Adherence in Certain Complication

Concerning diabetes, the survey participants identified various complications they were experiencing. Among the overall non-adherent group, the majority (27.0%) reported having high blood pressure, while a smaller percentage (15.8%) reported high cholesterol levels. The incidence of other complications was relatively lower, with 11.2% reporting eye damage, 8.7% reporting nerve damage, 3.6% reporting renal impairment, and 2.5% reporting obesity. However, there were no reports of diabetic foot or other symptoms.

**Figure 4:**
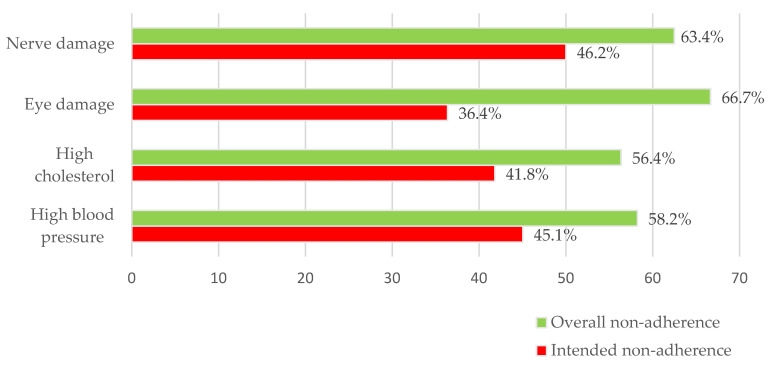
Percentage of non-adherence in certain complications of diabetes.

As shown in Figure 4, the survey results indicated that approximately 66.7% of respondents in the overall non-adherence group had eye damage or vision problems. In contrast, a higher percentage of respondents in the intended non-adherence group reported nerve damage or feeling numbness in the limbs, accounting for 46.2% of this group. This difference in the prevalence of complications between the two groups highlights the varying impact of non-adherence on different aspects of diabetic health.

It is important to note that eye damage appeared to be the complication with the greatest disparity between the intended and overall non-adherence groups. This suggests that non-adherence to treatment may have a more pronounced effect on the development or progression of eye-related complications compared to other complications.

### 3.7. Views of Patients Regarding the Accessibility and Skill of Services Provided by Local Pharmacists

**Figure 5:**
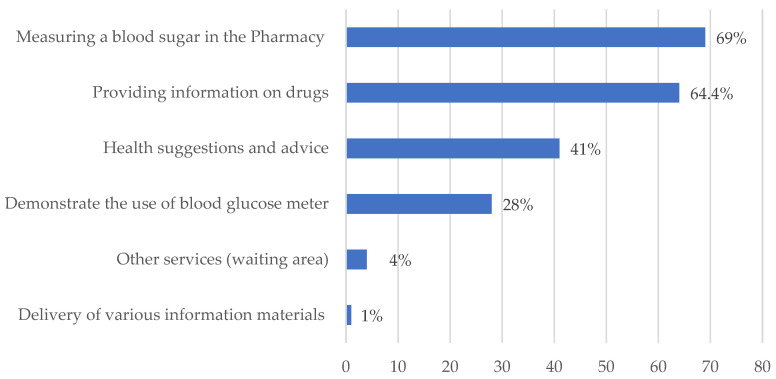
Patients’ perceptions of community-pharmacy diabetes services.

As can be seen in Figure 5, most respondents (64.40%) emphasized that community pharmacists are available to provide information related to treatments in the pharmacy, and 69% said that pharmacists who offered a blood test to monitor glucose levels treated them with great respect and politeness. Moreover, 41% said the pharmacist took sufficient time with them and gave them some sensible advice, such as diet recommendations and over-the-counter medications, and 28% also said the pharmacist seemed to be ready to provide them with clear answers to their questions and had shown them how to use the blood-glucose meter device with guidelines. However, only 1% of participants said that the delivery of various materials related to diabetes, such as brochures or up-to-date publications, was available in Latakian pharmacies.

### 3.8. Percentage of the Overall Population Satisfaction with the Services Provided by Their Local Pharmacy

**Figure 6:**
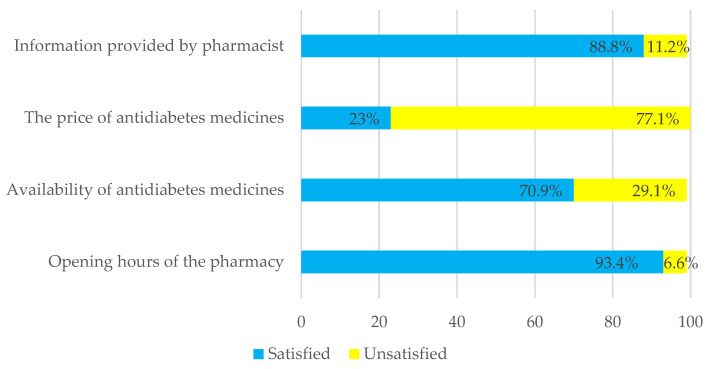
Satisfaction of diabetes patients among community-pharmacy services.

Patient satisfaction is a vital indicator of service quality in the healthcare and pharmacy sectors. In the survey results presented in Figure 6, we observed that patient responses regarding the cost and accessibility of their medications were inconsistent.

Approximately three-quarters (71%) of respondents expressed satisfaction with the availability of diabetes medications in pharmacies. This indicates that a majority of patients found it convenient to access the necessary medications for their condition, which is a positive aspect of the healthcare system.

Furthermore, a high percentage (88.8%) of respondents reported receiving clear instructions from pharmacists on how to use their medications. This demonstrates the valuable role of pharmacists in providing education and guidance to patients, ensuring they have a proper understanding of their drug therapy.

In terms of pharmacy opening times, the majority of patients (93.4%) expressed satisfaction. This suggests that the operating hours of pharmacies are generally suitable and accessible for patients, allowing them to obtain their medications conveniently.

However, when it came to diabetes drug prices, a significant proportion (77.10%) of patients reported dissatisfaction, particularly in the context of the current conflict in Syria. High drug prices can pose financial burdens and may affect patients’ ability to be adherent to their prescribed treatment plans.

Data are presented in Figure 6.

### 3.9. Percentage of Non-Adherent Patients’ Satisfaction with the Services Provided by Community Pharmacist

**Figure 7:**
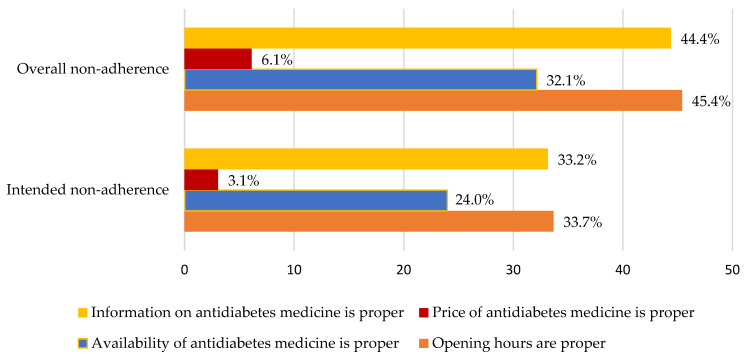
Percentage of non-adherent patients’ satisfaction with pharmacist services.

Referring to the survey results presented in Figure 7, among patients with diabetes in the overall non-adherent group, 44.4% of respondents expressed interest in and satisfaction with the information they received from community pharmacists regarding their medications. This indicates that a significant portion of patients in this group values the knowledge and guidance provided by pharmacists when it comes to their drug therapy.

On the other hand, only 6.1% of respondents in the overall non-adherent group reported being satisfied with the price of anti-diabetic drugs. This suggests that a majority of patients in this group find the cost of their medications to be unsatisfactory. The pattern observed in the intended non-adherent group is similar to that of the overall non-adherent group.

### 3.10. The Relationship between the Complexity of the Treatments and Patients’ Adherence

**Figure 8:**
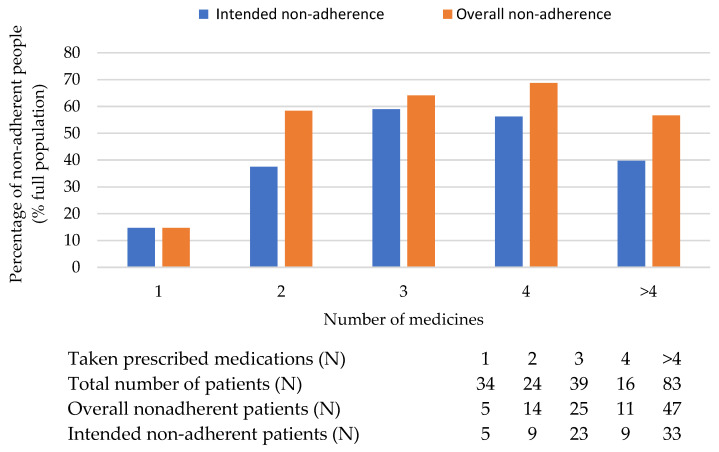
The relationship between the number of medicines that patients take and patient adherence. The corresponding table under the graph contains the raw data.

According to the survey findings, there was a correlation between the complexity of treatment regimens and patient adherence. The rate of therapeutic non-adherence tended to increase among patients who were required to take multiple tablets per day, particularly in the one, two, three, or four tablets range. However, interestingly, when the number of pills exceeded four, there was a decrease in the rate of non-adherence among patients in both the intended and overall non-adherence groups. This finding is illustrated in Figure 8.

The results suggest that the complexity of treatment regimens can be a significant barrier to patient adherence. Patients who have to manage multiple medications daily may face challenges in keeping up with their prescribed regimen, which can lead to non-adherence. However, as the number of pills increases beyond a certain threshold, it may indicate a shift towards more severe health conditions or a higher level of commitment to treatment, resulting in improved adherence.

## 4. Discussion

Based on our previous systematic review [21] various pharmaceutical care approaches seem successful and effective in increasing patient adherence, controlling glucose levels, and improving diabetes understanding. Furthermore, numerous studies have demonstrated that the community pharmacist, as a health promoter, can play a critical role in diabetes prevention [34,36,37,38,41,47,48,49].

Here, we performed a questionnaire survey to provide preliminary data on T2DM patients’ perceptions of community-pharmacy services in Syria with an emphasis on diabetic-patient therapeutic adherence. We reached 196 patients (87 females and 109 males) and asked about their perceptions of community-pharmacy services as well as their treatment behavior. From our study, it is clear that diabetic patients in Latakia, Syria are satisfied with the services offered by community pharmacies. Nevertheless, the cost of medications was the issue that affected participants most frequently.

Several similar studies have assessed the diabetic satisfaction and expectations towards community-pharmacy services world-wide [14,15,22,23,24,28,29,35,38,40,45,50,51].

It is important to maintain pharmaceutical care as it can be crucial in motivating patients to follow their treatment regimens and enhancing their quality of life. In our study, participants expressed positive opinions of community pharmacists and pharmacy services. The degree of patient satisfaction was shown to be influenced by several variables, including the patient’s age and educational background. Comparable studies showed that the positioning of the community pharmacy, the punctuality of services, and the expertise of the pharmacist were all significant determinants of customer satisfaction with community-pharmacy services [33,34,52]. Within our study, satisfaction with community-pharmacy services was also an important question. The patients were satisfied with the information that pharmacists provided and the opening hours of the pharmacy (88.8% and 93.4%, respectively). However, only 71% of patients were satisfied with the availability of the drugs. The rate of satisfaction with the medicine prices was only 23% (see Figure 6).

To assess the patients’ therapeutic behavior, we asked the participants in our survey several questions regarding the reasons that make patients non-adherent to treatment and divided them into two non-adherent groups. We assessed the patients’ adherence in our survey based on three factors: educational level, patients’ beliefs about the treatments, and the seriousness of the disease and emergence of other symptoms. In terms of educational level, we can note that the educational level was directly proportional to the patient’s commitment to treatments (see Figure 1). According to the response to our questionnaire, diabetes education needs to address these knowledge gaps with more precision.

Concerning the patients’ beliefs about the treatments, we can observe that the adverse drug effects can even lead to stopping or discontinuing the treatments (see Table 2). A study by Roborel de Climens et al. also showed the same effects [53]. As a result, maintaining ongoing adherence to the recommended drugs may be difficult in newly diagnosed patients with complex regimens. In our survey, around 15% of the participants indicated that the complexity of the treatments led to not taking their medicine as it has been prescribed. A similar study by Jimmy et al., showed that the complexity of the drug regimen negatively affected medication adherence [54].

However, adherence can be predicted by the patient’s disease state, and their awareness of its severity. Patients who are most severely ill with serious diseases may be at greatest risk for non-adherence to treatment, but this situation could make the patients more aware of the need to take their medicines in a proper way. In our survey, we made a comparison between non-adherent patients based on the number of pills that patient take (see Figure 8). DiMatteo et al. demonstrated that patients who had poorer health issues were more adherent to treatment [55]. The findings from our survey provide valuable insights into the potential factors contributing to patient non-adherence and suggest areas for improvement in healthcare delivery. Understanding how individuals perceive their disease is crucial for healthcare providers to support patients in preventing and managing their conditions effectively. To explore this aspect, we included a question in our survey that focused on the solutions patients would consider if their health deteriorated (see Figure 3).

The results indicate that a significant proportion (52.6%) of participants prefer visiting a general practitioner (GP) when experiencing health deterioration. This highlights the need for healthcare professionals, including community pharmacists, to enhance their diabetic education efforts and address the gaps in patient knowledge. By providing accurate and comprehensive information about diabetes management, community pharmacists can play a crucial role in reducing the burden on GPs and improving patient care.

To fill these gaps, it is essential to implement awareness campaigns and educational programs within the Syrian community. These initiatives should aim to educate the public about the roles and capabilities of community pharmacists in delivering patient-centered care and fostering meaningful interactions between patients and pharmacists. Emphasizing the importance of treatments, including lifestyle modifications such as adopting a better diet, without incurring additional costs, can be particularly impactful.

By addressing these needs and enhancing patient education, healthcare providers can improve patient adherence, reduce healthcare burdens, and effectively manage diabetes within the Syrian community. These efforts will contribute to a greater understanding of the disease and lead to more informed healthcare decisions and better health outcomes for patients.

Patients’ perspectives on the accessibility of pharmacist services were a crucial aspect explored in our study, and the specific examples are presented in Figure 5. The majority of participants generally rated each of these factors as being available. However, approximately 60% of participants expressed a lack of general health advice, including self-management support programs, information about drug–food interactions, and guidance on maintaining a healthy lifestyle. Furthermore, nearly all patients reported a lack of access to various information materials related to diabetes, such as brochures, up-to-date publications, online resources, and leaflets. These findings highlight the need for awareness initiatives and informational campaigns within the Syrian community to educate patients about treatment adherence and enhance the community pharmacists’ skills in delivering satisfactory diabetes care.

Providing informative brochures can be an effective and low-cost approach to delivering information that may improve medication adherence. Such materials can serve as valuable resources for patients, providing them with essential knowledge about their condition and treatment options. Research conducted by Caetano et al. supports the positive impact of interventions using leaflets distributed in basic healthcare settings, particularly among younger individuals with limited research exposure, in promoting medication adherence. Therefore, addressing the information gaps and enhancing the delivery of pharmacist services are vital for improving patient satisfaction and treatment adherence among individuals with diabetes in the Syrian community. Awareness initiatives, such as distributing informative brochures, and conducting informational campaigns, can play a significant role in achieving this goal. By empowering patients with accurate and accessible information, healthcare providers can support better self-management and overall health outcomes for individuals with diabetes [56].

Future studies with a larger sample size and conducted over a longer period are needed to confirm the importance of pharmacy services in diabetes care. To improve patient satisfaction with the pharmacists’ role as health care providers and to improve the professional image of the pharmacy, we could recommend appointing one pharmacist in each community pharmacy for only consultations and medical-history reviews.

### Limitation

Most importantly, probability sampling was not achieved; hence, not all types of Syrian patients may be covered well by the study. Furthermore, the response rate was also rather low, and only a small number of patients were considered which might hinder how far the results can be accepted as a general feature of Latakian diabetic patients. A future study examining community pharmacists’ viewpoints and experiences in improving diabetes therapeutic adherence would provide a more comprehensive review and examine other pharmacist strategies in diabetes care. Because of these limitations, only basic statistics were provided, and further analysis was not conducted.

## 5. Conclusions

Our survey highlighted the positive perception of T2DM patients toward the services provided by community pharmacies. These patients also value the education and counseling provided by pharmacists on medication management and patients’ therapeutic adherence to avoid treatment interruption, which can be acquired by comprehensive and coordinated care for T2DM patients.

## Figures and Tables

**Table 1:** Characteristics of the patients.

Type 2 Diabetes Respondents	Number (%)
**Gender**	
Male	109 (55.6%)
Female	87 (44.4%)
**Age**	
18–30	3 (1.5%)
31–45	30 (15.3%)
46–60	86 (43.9%)
61–70	77 (39.9%)
**Place of residence**	
Country side	30 (15.3%)
City center	147 (75%)
Village	19 (9.7%)
**Level of education**	
Elementary	36 (18.4%)
High school (intermediate)	98 (50%)
College or higher	62 (31.6%)
**Duration of diabetes disease**	
Less than 6 months	22 (11.2%)
More than 6 month	174 (88.8%)
**Family member had diabetes**	
Yes	144 (73.5%)
No	52 (26.5%)
**Any complications of diabetes**	
Yes	122 (62.2%)
No	74 (37.8%)

**Table 2:** Patients’ adherence to prescribed medicines (GP: general practitioner).

Questions	Yes (%)	No (%)
Daily medication is a problem for many. Is this a problem for you in your everyday life?	71 (36.2%)	125 (63.8%)
Do you often forget to take your medication?	49 (25%)	147 (75%)
Is your doctor advising you to take anti-diabetic medicines for your diabetes?	195 (99.5%)	1 (0.5%)
Have you stopped taking your diabetes medicine(s) because you are suspected of feeling ill from taking this medicine(s)?	25 (12.8%)	171 (87.7%)
Have you stopped or discontinued your diabetes medication(s) because your condition has not improved and you are discouraged from taking this medication(s)?	37 (18.9%)	159 (81.1%)
When you feel that your diabetes is in balance, do you stop taking the medicine(s) for your diabetes?	43 (21.9%)	153 (78.1%)
Do you stop taking your diabetes medicine(s) if your friend/relative/neighbor is taking the same medicines as you and has experienced any side effects?	35 (17.9%)	161 (82.1%)
Is it a problem to ask GP about the drug therapy of diabetes?	9 (4.6%)	187 (96.9%)
Is it a problem to ask the pharmacist about the drug therapy of diabetes?	19 (9.7%)	177 (90.3%)

## Data Availability

Not applicable.

## Supplementary Materials

The following supporting information can be downloaded at: https://www.mdpi.com/article/10.3390/healthcare11121720/s1, Survey: perceptions of T2DM patients towards community pharmacy services.
