# Supplementary material for: Type 2 Diabetes Patients’ Views of Local Pharmacists and Fulfilment with Pharmaceutical Diabetes Care in Syria’s Latakia Governorate: An Online Survey Research
Source: Healthcare (Basel). 2023 Jun 12;11(12):1720. doi: 10.3390/healthcare11121720 (PMC10298587; doi:10.3390/healthcare11121720)
Supplement: Supplementary file 1 [file healthcare-11-01720-s001.zip › healthcare-2421426-supplementary.pdf]

Survey: perceptions of T2DM patients towards community pharmacy services

Dear Ladies and Gentlemen,

My name is Sarah Al Assaf, a Ph.D. candidate in Pharmaceutical Science Doctoral School of Pharmacy Administration and drug development at the Semmelweis University in Hungary. I am conducting a study about the Effect of Community Pharmacy services in increasing the therapeutic adherence of T2DM patients in Latakia,Syria
We cordially invite you to take part in a survey on T2DM patients perceptions towards the community pharmacy services. Please only complete the questionnaire if you are diagnosed with T2DM and are taking any prescription medication (such as tablets or capsules). However, if you are already using insulin or other injectable medicines for your diabetes, and a pregnant woman please do not fill in the questionnaire. If you are taking other medicines for other illnesses, you can still fill the questionnaire!
The information obtained in this survey is just for scientific purposes only and the participants remains anonymous.
Your contribution is very important to achieve the study objectives.
Thank you for your cooperation!
If you have any questions about this study, feel free to contact me at
sarah.assaf@phd.semmelweis.hu

سيداتي وسادتي،

اسمي الصيدلانية سارة العساف ، طالبة دكتوراه في قسم الصيدلة السريرية وتطوير الأدوية في كلية الصيدلة في جامعة سيميلويس, المجر. أقوم بإجراء دراسة حول تأثير خدمات الصيدلة المجتمعية على زيادة الالتزام العلاجي لمرضى السكري من النوع 2 في سوريا.
لطفا ادعوك (ي) للمشاركة في هذا الاستبيان لأخذ فكرة واضحة عن تصورات مرضى السكري من النوع الثاني اتجاه الخدمات المتوفرة في الصيدليات العامة . يرجى إكمال الاستبيان فقط إذا تم تشخيصك بمرض السكري وكنت تتناول دواءً موصوفًا (مثل حبة أو كبسولة) لعلاج مرض السكري.
إذا كنت تتناول بالفعل الأنسولين أو أدوية أخرى قابلة للحقن، فيرجى عدم ملء الاستبيان. إذا كنت تتناول أدوية أخرى لأمراض أخرى ، فلا يزال بإمكانك ملء الاستبيان!
المعلومات التي تم الحصول عليها في هذا الاستبيان هي للأغراض العلمية فقط وسيكون هذا الاستبيان على قدر من الخصوصية.
مساهمتك مهمة للغاية.
شكرا جزيلا لتعاونكم!

إذا كان لديك أي أسئلة حول الدراسة ، فلا تتردد في الاتصال بي
E-mail: sarah.assaf@phd.semmelweis.hu

الجزء الأول: البيانات الشخصية

Part 1: Demographic patients' characteristics

1.Your gender (الجنس): □ Male (ذكر) □ Female (انثى)

2.Your age (العمر):

□ 18-30

□ 31-45

□ 46-60

□ 61-75

3. our place of residence (مكان السكن) :

□ country side (الريف)

□ city center (وسط المدينة)

□ village (قرية)

4. Your highest level of education: (مستوى التعليم)

□ elementry (تعليم ابتدائي)

□ intermediate (تعليم متوسط)

□ higher education (تعليم عالى)

Part 2: Patient therapeutic behavior (سلوك المريض العلاجي)

5. How many prescription drugs are you taking in total? كم عدد الأدوية الموصوفة التي تتناولها إجمالاً؟

□ 1

□ 2

□ 3

□ 4

□ > 4

6. Daily medication is a problem for many. Is this a problem for you in your everyday life? الأدوية اليومية مشكلة بالنسبة للكثيرين. هل هذه مشكلة بالنسبة لك في حياتك اليومية؟	Yes (نعم)	No (كلا)
7. Do you often forget to take your medication? هل تنسى كثيرًا تناول أدويتك؟	Yes (نعم)	No (كلا)

8. How often is it difficult to remember to take your medicine (s)? كم مرة يكون من الصعب تذكر تناول الدواء (الأدوية) الخاص بك؟

□ Always دائما

□ Often غالبا

□ Sometimes احيانا

□ Rarely نادرا

□ Never أبدا

9. Is your doctor advising you to take your medicines for your diabetes? هل ينصحك طبيبك بتناول الأدوية المضادة لمرض السكر؟	Yes (نعم)	No (كلا)

10. Have you stopped or stopped taking your diabetes medicine (s) because you are suspected of feeling ill from taking this medicine (s)? هل توقفت عن تناول دواء (أدوية) السكري بسبب الاشتباه في شعورك بالمرض بسبب تناول هذا الدواء (الأدوية)؟	Yes (نعم)	No (كلا)
11. Have you stopped or discontinued your diabetes medication (s) because your condition has not improved and you are discouraged from taking this medication (s)? هل توقفت عن تناول دواء (أدوية) السكري لأن حالتك لم تتحسن ولايوجد احد يُشجعك على تناول هذا الدواء (الأدوية)؟	Yes (نعم)	No (كلا)
12. When you feel that your diabetes is in balance, do you stop taking the medicine (s) for your diabetes? عندما تشعر أن مرض السكري لديك في حالة توازن ، هل تتوقف عن تناول دواء (أدوية) ؟	Yes (نعم)	No (كلا)
13. Do you stop taking your diabetes medicine (s) if your friend / relative / neighbor is taking the same medicines as you and has experienced any side effects? هل تتوقف عن تناول دواء (أدوية) السكري إذا كان صديقك / قريبك / جارك يأخذ نفس الأدوية التى تتناولها وتعرض لأية آثار جانبية؟	Yes (نعم)	No (كلا)
14. Is it a problem for you to ask the GP about the drug therapy for their diabetes? هل تواجه مشكلة في سؤال الطبيب العام عن العلاج الدوائي لمرض السكري؟	Yes (نعم)	No (كلا)
15. Is it a problem for you to ask the pharmacist questions about the drug therapy for your diabetes? هل تواجه مشكلة فى طرح أسئلة حول العلاج الدوائي لمرض السكري فى الصيدلية؟	Yes (نعم)	No (كلا)
16. Please indicate the most common reasons why you may not be taking your diabetes medicine (s) as recommended by your doctor or pharmacist.<br>(You can enter more than one answer!)<br>يرجى الإشارة إلى الأسباب الأكثر شيوعًا التي قد تجعلك لا تتناول أدوية السكري الخاصة بك على النحو الموصى به من قبل طبيبك أو الصيدلي (يمكنك إختيار أكثر من إجابة واحدة !)<br>□ Overall, it is too complicated to take this (these) medicine (s) بشكل عام ، من الصعب جدًا تناول هذه الأدوية<br>□ Lack of family support عدم وجود دعم الأسرة<br>□ Lack of any motivation عدم وجود أي دافع لتناول الدواء		

□ I have been taking this medicine (s) for too many years (الأدوية) لقد كنت أتناول هذا الدواء لسنوات عديدة<br>□ My meals are affected by taking this medicine (s) (الأدوية) تتأثر وجباتي بتناول هذا الدواء<br>□ Fear of side effects (e.g. hypoglycemia) الخوف من الآثار الجانبية (مثل نقص السكر في الدم)<br>□ I always forget to take this medicine (s) (الأدوية) أنسى دائمًا تناول هذا الدواء<br>□ I feel this (these) drug (s) are not effective for me أشعر أن هذه الادوية ليست فعالة بالنسبة لى		
17. In total, how many tablets or capsules should you take in just one day for your diabetes? (Whether from just one type of medicine, or even from several types of medicine combined!) كم عدد الأقراص أو الكبسولات التي يجب أن تتناولها في يوم واحد فقط لمرض السكري لديك؟ (سواء من نوع واحد فقط من الأدوية ، أو حتى من عدة أنواع من الأدوية مجتمعة !)<br>□ only one<br>□ two kinds<br>□ three kinds<br>□ more than three kinds<br>□ I do not know		
18. When should you only take medication for your diabetes in one day? متى يجب أن تتناول الدواء ؟<br><br>□ only in the morning صباحا فقط<br>□ only in the afternoon ظهرا فقط<br>□ only in the evening في المساء<br>□ morning and evening صباحا و مساءا<br>□ morning, noon and evening صباحا, ظهرا و مساءا		
Part 3: Diabetes knowledge الجزء الثالث: مرض السكري		
19. When was it found that you were diabetic? منذ متى تم تشخيصك بمرض السكري؟<br>□ less than 6 months اقل من 6 اشهر<br>□ more than 6 months اكثر من 6 اشهر		
20. Was there a diabetic family member in the family? هل يوجد فرد في الأسرة مصاب بمرض السكري؟	Yes (نعم)	No (كلا)
21. Do you have a complication of your diabetes? هل لديك مضاعفات لمرض السكري الخاص بك؟ أي هل لديك شكوى، او مرض ظهر بعد تشخيص الاصابة بمرض السكري؟ سواء كان مع أو بدون تقرير طبي<br>*do you have a complaint, an illness, that appeared after the onset of diabetes, Whether with or without a medical report.*	Yes (نعم)	No (كلا)

22. If you have a complication, what is it like? (Multiple answers possible!)<br>*Only mark a complaint if it occurred after the onset of diabetes.* اذا كانت لديك مضاعفات ، ماهى هذه المضاعفات؟ ضع علامة على شكوى فقط إذا حدثت بعد التشخيص بالاصابة بمرض السكري (يمكن اختيار اكثرمن اجابة واحدة!)<br>□ Eye damage or vision problems تلف في العين أو مشاكل في الرؤية<br>□ Renal impairment or complaints القصور الكلوي<br>□ High blood pressure ارتفاع ضغط الدم<br>□ High cholesterol or triglyceride levels ارتفاع الكوليسترول أو الدهون الثلاثية<br>□ Feeling nerve damage or numbness in the limbs الشعور بتلف في الأعصاب أو تنميل في الأطراف<br>□ Diabetic foot القدم السكرية<br>□ Obesity or weight gain السمنة أو زيادة الوزن<br>□ I have no diabetec symptoms (other symptoms for example: headache) اعراض اخرى
23. What are you doing to ensure that you do not develop complications of diabetes in the future or that your current health condition worsens?<br>*(Multiple answers available!)* ما الذي تفعله للتأكد من عدم تعرضك لمضاعفات مرض السكري فى المستقبل أو أن حالتك الصحية الحالية تزداد سوءًا؟ (يمكن اختيار اكثرمن اجابة واحدة!)<br><br>□ I keep an eye on my weight مراقبة الوزن<br>□ Restricting and reducing high fat foods during my meals تقليل الأطعمة الغنية بالدهون<br>□ Restricting and reducing sugar intake during my meals تقليل تناول السكر<br>□ Regular exercise, physical activity ممارسة الرياضة بانتظام<br>□ I go to the doctor regularly أذهب إلى الطبيب بانتظام<br>□ I do nothing لا أفعل شيئًا<br>□ I don't know what I could do لا أعلم ما الذي يمكنني فعله
Part 4): Pharmacy services: الجزء الرابع: خدمات الصيدلية لمرضى السكري<br>Below, we are curious about your level of satisfaction with pharmacy services for your diabetes.<br>Next to the statements, check the option you agree with.

24. The opening hours of the pharmacy I visit most often ساعات عمل الصيدلية التي أزورها!	Satisfied (جيدة)	Unsatisfied (غير جيدة)
25. Availability of the drug (s) prescribed fo me for my diabetes in the pharmacy توافر الأدوية الموصوفة لمرض السكري فى الصيدلية	Satisfied (جيدة)	Unsatisfied (غير جيدة)
26. The price of my drug (s) for my diabetes. سعر الدواء (الأدوية) الخاصة بمرض السكري	Satisfied (جيدة)	Unsatisfied (غير جيدة)
27. In general, information on the use of medications provided by pharmacy staff	Satisfied (جيدة)	Unsatisfied (غير جيدة)

for my diabetes (e.g., dosage, side effects, expected efficacy) المعلومات عن كيفية استخدام الأدوية التى يقدمها طاقم الصيدلية لمرضى السكري (مثل الجرعة والآثار الجانبية والفعالية المتوقعة)		

28. What kind of diabetes-related service have you ever encountered in public pharmacies?ما نوع الخدمة المتعلقة بمرض السكري و المتوفرة في الصيدليات العامة
(Multiple answers available!) (يمكنك اختيار اكثر من اجابة واحدة !)

- □ Information on how to take your medicine (s) for diabetes (e.g., what, why and how should I take it?) تقديم معلومات حول كيفية تناول الأدوية الخاصة بك (على سبيل المثال ، ماذا ولماذا وكيف يجب أن أتناوله؟)
- □ Health suggestions and advice on diabetes other than taking medication (e.g., diet, exercise, etc.) تقديم قتراحات ونصائح صحية حول مرض السكري بخلاف تناول الأدوية (مثل النظام الغذائي والتمارين الرياضية وما إلى ذلك)
- □ Measuring blood sugar in the pharmacy قياس سكر الدم بالصيدلية
- □ Demonstrate the use of the device when purchasing a blood glucose meter تقديم معلومات عن كيفية استخدام الجهاز عند شراء جهاز قياس السكر في الدم
- □ Delivery of various information materials related to diabetes (e.g., publications, online access, distribution of leaflets, etc. زيادة تثقيف المريض عن طريق تقديم مواد إعلامية متنوعة تتعلق بمرض السكري (مثل المطبوعات ، والوصول عبر الإنترنت ، وتوزيع المنشورات ، وما إلى ذلك)
- □ Other services (waiting area, counselling service) خدمات اخرى (الانتظار والاستشارة الدوائية)

Thank you for completing the questionnaire! Your cooperation means a lot to us!

شكرا لكم لاستكمال الاستبيان! تعاونكم يعني الكثير بالنسبة لنا
